# Prognostic Value of Left Ventricular Myocardial Strain Parameters Derived from Cardiac Magnetic Resonance Feature Tracking Technique in Light-Chain Cardiac Amyloidosis Patients: A Pilot Study

**DOI:** 10.31083/j.rcm2511400

**Published:** 2024-11-14

**Authors:** Rile Nai, Jia Liu, Kai Zhao, Shuai Ma, Wei Ma, Jiangkai He, Shasha Xu, Jianxiu Lian, Wei Li, Jianxing Qiu

**Affiliations:** ^1^Department of Radiology, Peking University First Hospital, 100034 Beijing, China; ^2^Department of Cardiovascular, Peking University First Hospital, 100034 Beijing, China; ^3^Clinical & Technical Support, Philips Healthcare, 100016 Beijing, China

**Keywords:** light-chain cardiac amyloidosis (AL-CA), cardiac magnetic resonance feature tracking (CMR-FT), myocardial strain, biomarker, prognostic

## Abstract

**Background:**

Previous research on the prognostic implications of left ventricular myocardial strain using cardiac magnetic resonance feature tracking (CMR-FT) in light-chain cardiac amyloidosis (AL-CA) has shown promising potential. This study aimed to evaluate the prognostic significance of global and segmental left ventricular myocardial strain in AL-CA patients, specifically analyzing the American Heart Association's 16 segments.

**Methods:**

A total of 75 consecutive patients (50 men, mean age: 55.6 ± 10.0 years) who underwent CMR examination with histologically confirmed systemic AL-CA were retrospectively enrolled between January 2014 and November 2022. Both global and segmental myocardial strain and the American Heart Association’s 16 segments were quantified using CMR-FT on the steady-state free precession (SSFP) cine sequence. A comparative analysis was conducted between survivors and non-survivors based on the defined endpoint. Student *t*-test or Mann–Whitney U, receiver operating characteristic curve, Kaplan–Meier event-free survival curve, and Cox proportional hazards regression were used. Significance was set at *p* < 0.05.

**Results:**

Following a median follow-up of 34 months, 16 out of 75 patients experienced mortality events. B-type natriuretic peptides (BNP) (*p* < 0.001), global radial strain (RS_global_) (*p* = 0.033), and RS in the basal inferior segment (RS_bas-inferior_) (*p* = 0.025) remained significant as independent predictors of all-cause mortality. The cut-off values were identified as 24.97% for RS_global_, and 20.97% for RS_bas-inferior_. Kaplan–Meier survival curves revealed significantly reduced event-free survival for individuals in the lower cut-off groups for RS_global_ and RS_bas-inferior_ (*p* = 0.013, *p* < 0.001, respectively).

**Conclusions:**

Radial strain for the global and the basal inferior segment may prove valuable for risk stratification in patients with AL-CA.

## 1. Introduction

Amyloidosis is a systemic disorder characterized by the deposition of insoluble amyloid in various tissues and organs [1], with light-chain amyloidosis emerging as the predominant form and capable of affecting multiple organs [2]. Cardiac involvement is prevalent in approximately 50% of cases and stands as a pivotal factor influencing patient survival [3]. Consequently, the early detection and ongoing monitoring of cardiac involvement hold significant potential for improving outcomes in individuals with light-chain cardiac amyloidosis (AL-CA).

Serum cardiac biomarkers such as troponin (Tn), N-terminal pro-B-type natriuretic peptide (NT-proBNP), and B-type natriuretic peptide (BNP) serve as indicators of cardiac involvement and can play a pivotal role in assessing prognosis in AL-CA [4, 5]. ﻿Non-Doppler echocardiographic parameters, including increased left ventricular (LV) wall thickness and decreased fractional shortening, are recognized as independent predictors of cardiac mortality in AL amyloidosis. [6]. Recently, cardiac magnetic resonance (CMR) has emerged as a valuable tool for both diagnosing cardiac amyloidosis and predicting patient outcomes. Among the various techniques, cardiac magnetic resonance ﻿feature tracking (CMR-FT) has gained prominence due to its simplicity and reliability in quantifying LV strain using steady-state free precession (SSFP) sequences [7, 8]. The calculation of strains in three directions of the ventricle is now a significant parameter in evaluating patients with heart transplantation and heart failure [9, 10].

A growing body of research has consistently demonstrated that assessing myocardial strain holds substantial promise in providing prognostic insights for patients with AL-CA [11, 12, 13]. While the assessment of LV function primarily relies on the measurement of left ventricular ejection fraction (LVEF), which reflects changes in LV volume, the evaluation of myocardial strain offers insights into myocardial deformation and has demonstrated high sensitivity in assessing myocardial function [14, 15]. Notably, the myocardial strain has been shown to have superior prognostic value over LVEF in specific clinical contexts involving patients with AL-CA [16, 17]. However, notable advancements have been made in utilizing strain techniques to assess the prognosis of patients with cardiac amyloidosis; nevertheless, research specifically addressing the prognostic implications of myocardial strain in different myocardial segments remains limited [18].

This study primarily aimed to evaluate changes in global, segmental, and American Heart Association’s 16-segment LV myocardial strain using CMR-FT on the SSFP cine sequence and to further assess these strain measurements as potential prognostic markers in patients diagnosed with AL amyloidosis.

## 2. Methods

### 2.1 Study Population

This study was conducted in accordance with the Declaration of Helsinki (2013 revision). Approval for this retrospective study was granted by the Ethics Committee on Scientific Research of our hospital; meanwhile, individual consent for a retrospective analysis was waived.

**Fig. 1.**
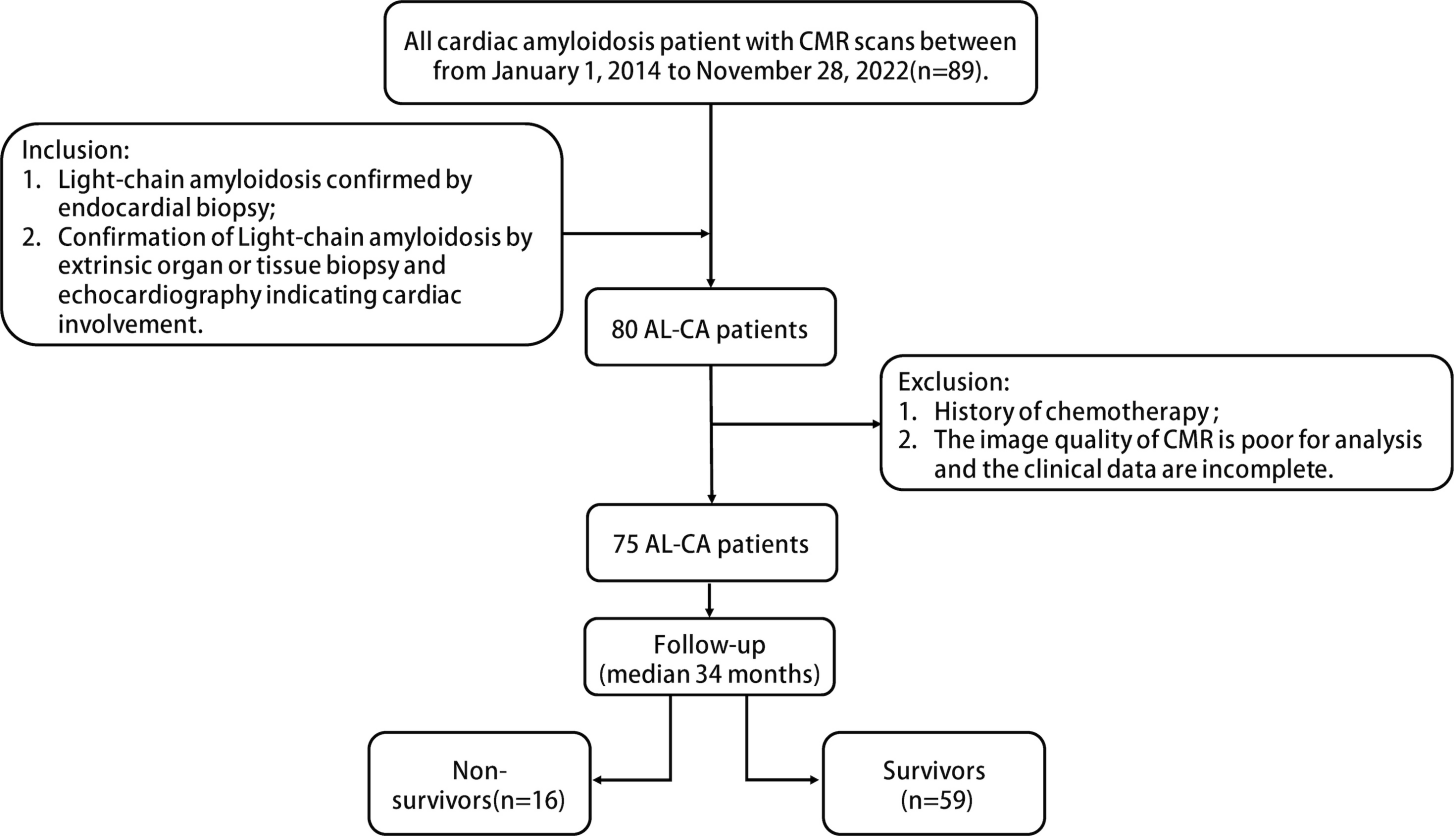
**Flowchart of the patient selection process in the study according to inclusion and exclusion criteria**. CMR, cardiac magnetic resonance; AL-CA, light-chain cardiac amyloidosis.

In this retrospective observational study, the magnetic resonance (MR) images of 89 consecutive patients who had undergone cardiac MR imaging for either clinically suspected or confirmed cardiac amyloidosis between January 2014 and November 2022 were acquired from the picture archiving and communication system (PACS). A diagnosis of AL-CA was established through a biopsy of subcutaneous fat or an involved organ demonstrating Congo red staining with apple green birefringence, detection of monoclonal protein in serum or urine, and/or identification of monoclonal plasma cells in the bone marrow. Furthermore, cardiac amyloidosis was defined as LV wall thickness exceeding 12 mm without another known cause, confirmed by echocardiography, diffuse late gadolinium enhancement on CMR imaging, or both [19]. According to the Boston University staging system, patients were classified as follows: (1) Stage I: Both BNP and cardiac troponin-I (cTnI) are below their thresholds (BNP <81 pg/mL and cTnI <0.1 ng/mL); (2) Stage II: One biomarker is above its threshold (BNP ≥81 pg/mL or cTnI ≥0.1 ng/mL), while the other is below; (3) Stage III: Both biomarkers are above their thresholds (BNP ≥81 pg/mL and cTnI ≥0.1 ng/mL) [20]. The exclusion criteria were a history of chemotherapy, poor image quality, and incomplete clinical data. The specific standards for the poor image quality were as follows: (1) Motion artifacts: Images with significant blurring or ghosting due to patient movement. (2) Low signal-to-noise ratio: Images where the myocardial borders were not distinguishable due to excessive noise. (3) Incomplete coverage: Images that did not fully recognize the entire left ventricular myocardium. The flow chart is shown in Fig. 1.

### ﻿2.2 Follow-up

﻿The primary endpoint of this study was defined as all-cause mortality, which was assessed through the patient’s hospital chart or telephone interviews conducted by an investigator who remained unaware of the patients’ clinical and cardiac MR imaging data. The follow-up period for the patients was extended until September 2023, and the duration between the date of cardiac MR imaging and the event of death was recorded as the time to event.

### 2.3 Cardiac MR Imaging Protocol

All patients underwent magnetic resonance imaging (MRI) scanning using a 3.0T scanner (Philips Ingenia (6.1.571.0, Philips Medical Systems, Best, The Netherlands), GE Discovery MR750 (DV24, GE Healthcare, Milwaukee, WI, USA), SIEMENS MAGNETOM Prisma (VE11E, Siemens Healthineers, Erlangen, Germany)) and a 1.5T scanner (SIEMENS MAGNETOM Aera (VE11C, Siemens Healthineers, Erlangen, Germany)) with a cardiac or abdominal coil. Images were acquired with retrospective electrocardiogram (ECG) gating during end-expiratory breath holding. ﻿SSFP cine images were obtained in consecutive short axes covering the LV and the long axis (two-, three-, and four-chamber views) according to the standardized protocol [21].﻿ The detailed imaging parameters for each scanner are provided in **Supplementary Table 1**.

### 2.4 CMR Imaging Analyses

For analysis, CMR images were imported into CVI42 v5.14.2 software (Circle Cardiovascular Imaging Inc., Calgary, AB, Canada). Two experienced radiologists performed the measurements, each with 4 and 5 years of experience in CMR diagnosis. To analyze the short-axis images, the software automatically detected the endocardial and epicardial contours of the left ventricle during both the diastolic and systolic phases. Subsequently, manual adjustments were used to ensure accurate delineation layer by layer. General CMR parameters and CMR-FT analyses were conducted. The general CMR parameters were exported, including the left ventricular end-diastolic volume index (LVEDVi), left ventricular end-systolic volume index (LVESVi), and LVEF.

As for strain indicators, global strain parameters of the left ventricle, such as radial strain (RS), circumferential strain (CS), and longitudinal strain (LS), were obtained first. Secondly, the segmental strain parameters of the left ventricle were measured in different regions of the myocardium, including the basal, middle, and apical segments. The segmental strain parameters were further assessed in 16 segments of the myocardium using the American Heart Association’s 16-segment model [22].

### ﻿2.5 Intra- and Interobserver Variability

﻿Intra- and interobserver variabilities in strain values were evaluated in 20 randomly selected AL amyloidosis patients using the intraclass correlation coefficient. Interobserver variability was assessed by two independent investigators using the same image set. Intraobserver variability was evaluated on the identical image set by one investigator one month later.

### 2.6 Statistics

Categorical variables are presented as the count and percentage; parametric continuous variables as the mean ± standard deviation (SD); non-parametric continuous variables as the median and interquartile range (IQR). Group differences in non-parametric continuous variables were assessed using the Mann–Whitney U test. Kaplan–Meier curves were utilized to estimate survival distribution over follow-up duration. The association of clinical, imaging, and serological parameters with outcomes was evaluated using both univariate and multivariate Cox proportional-hazards regression models. For Cox regression analysis, segmental strain values were transformed into categorical variables based on predefined cut-offs, where values above the cut-off were categorized as high and below as low. Receiver operating characteristic (ROC) curves were constructed to determine optimal cut-off values for endpoints, and the DeLong method was employed to compare the area under the curve (AUC). Statistical significance was defined as *p* < 0.05. Statistical analyses were conducted using SPSS version 26 (IBM, Armonk, NY, USA) and GraphPad Prism 9 (GraphPad, San Diego, CA, USA).

## 3. Results

### ﻿3.1 Baseline Characteristics

Among the cohort of 89 patients, 80 received a diagnosis of AL-CA. One individual faced exclusion due to a history entailing chemotherapy, while four others were omitted owing to inadequate CMR image quality and incomplete clinical data. Consequently, the refined study population comprised 75 patients (50 men, with an average age of 56 ± 10 years). Over a median follow-up period of 34 months (ranging from 24 to 41 months), 16 patients (21%) experienced mortality events. The baseline clinical characteristics of the entire study population, categorized into survivors and non-survivors, are presented in Table 1. In the comparative analysis between these groups, survivors exhibited similar age, gender, body mass index (BMI), and blood pressure when compared to non-survivors (all *p* > 0.05). Non-survivors demonstrated significantly elevated levels of Modification of Diet in Renal Disease (MDRD), BNP, cTnI, and Boston University (BU) stage when compared to survivors (*p* = 0.043, *p* < 0.001, *p* < 0.001, and *p <* 0.001, respectively).

**Table 1.** **﻿Demographics and cardiovascular magnetic resonance (CMR) data of patients with amyloidosis (n = 75), with and without reaching the endpoints during the follow-up**.

Variables		All	Survivors	Non-survivors	*p*-value
		(n = 75)	(n = 59)	(n = 16)	
Clinical data					
	Age (years)	55.6 ± 10.0	54.7 ± 10.8	58.8 ± 5.6	0.290
	Male gender, n (%)	50 (67%)	38 (64%)	12 (75%)	0.555
	BMI (kg/m^2^)	23.5 ± 2.6	22.9 ± 3.5	22.7 ± 2.5	0.113
	Systolic BP (mmHg)	114.9 ± 14.4	116.7 ± 17.7	112.7 ± 13.2	0.755
	Diastolic BP (mmHg)	72.0 ± 10.9	72.4 ± 12.0	72.8 ± 9.5	0.379
Survival times (months)		34 (24–41)	28 (25–45)	19 (12–35)	0.033
Laboratory data					
	MDRD (mL/min/1.73 m^2^)	88.1 ± 23.6	91.5 ± 21.5	75.2 ± 27.1	0.043
	﻿Troponin I (µg/L)	0.1 ± 0.5	0.1 ± 0.5	0.1 ± 0.1	<0.001
	BNP (pg/mL)	416.2 ± 617.8	223.1 ± 226.5	1128.0 ± 998.8	<0.001
BU stage, n (%)					<0.001
	I	17 (23%)	17 (29%)	0	
	II	47 (63%)	37 (63%)	10 (63%)	
	III	11 (15%)	5 (8%)	6 (38%)	
CMR data					
	LVEDVi (mL/m^2^)	63.6 ± 16.4	65.1 ± 17.4	58.1 ± 10.4	0.154
	LVESVi (mL/m^2^)	31.7 ± 10.8	31.6 ± 11.3	32.0 ± 9.1	0.873
	LVEF (%)	50.6 ± 9.8	52.0 ± 9.9	45.7 ± 8.1	0.014
	RS_global_ (%)	27.6 ± 11.3	29.1 ± 11.7	22.3 ± 8.3	<0.001
	RS_basal_ (%)	29.0 ± 15.2	31.9 ± 15.4	18.3 ± 8.0	0.029
	RS_middle_ (%)	22.8 ± 9.3	24.1 ± 9.6	17.9 ± 6.1	<0.001
	RS_apical_ (%)	38.6 ± 27.7	38.4 ± 24.1	39.3 ± 39.2	0.903
	CS_global_ (%)	–15.7 ± 4.3	–16.1 ± 4.1	–13.9 ± 4.4	<0.001
	CS_basal_ (%)	–14.5 ± 5.3	–15.2 ± 5.5	–11.9 ± 4.3	<0.001
	CS_middle_ (%)	–15.7 ± 5.6	–16.1 ± 5.8	–14.3 ± 4.9	0.014
	CS_apical_ (%)	–17.3 ± 4.9	17.5 ± 5.0	–16.5 ± 4.7	0.499
	LS_global_ (%)	–9.8 ± 5.0	–10.1 ± 5.1	–8.9 ± 4.3	0.112
	LS_basal_ (%)	–8.3 ± 6.4	–8.6 ± 6.6	–7.1 ± 5.6	0.023
	LS_middle_ (%)	–9.0 ± 7.0	–9.3 ± 7.4	–8.1 ± 5.6	0.903
	LS_apical_ (%)	–12.0 ± 4.0	–12.5 ± 3.9	–10.5 ± 4.4	0.002

Note: BMI, body mass index; BP, blood pressure; BNP, B-type natriuretic peptides; MDRD, ﻿Modified Diet in Renal Disease, (MDRD equation (Glomerular filtration rate (GFR) = 175 × standardized × serum creatinine (S_cr_) ^-1.154^ × age ^-0.203^ × 1.212 [if black] × 0.742 [if female])); BU, Boston University; LVEDVi left ventricular end-diastolic volume index; LVESVi, left ventricular end-systolic volume index; LVEF, left ventricular ejection fraction; RS, radial strain; CS, circumferential strain; LS, longitudinal strain.

### 3.2 General CMR Parameters and Strain Parameters

The comprehensive assessment of cardiac parameters, both general and strain-related, obtained from CMR cine is presented in Table 1. Survivors and non-survivors exhibited similarities in LVEDVi and LVESVi, with no statistically significant differences between the two groups (*p* = 0.154, *p* = 0.873, respectively). However, a notable distinction emerged in LVEF, which was significantly lower in the non-survivors than in the survivors group (*p* = 0.014).

Turning to the LV global strain parameters assessment, both global radial strain and circumferential strain exhibited a substantial change in the non-survivor group compared to the survivors (RS_global_, *p* < 0.001; CS_global_, *p* < 0.001). In terms of LV segmental strain parameters, significant differences were discerned between survivors and non-survivors in the basal segments of radial strain, circumferential strain, and longitudinal strain (RS_basal_, *p* = 0.029; CS_basal_, *p* < 0.001; LS_basal_, *p* = 0.023). Moreover, significant variations were noted in the middle segments of radial strain, circumferential strain, and apical segments of longitudinal strain between the two groups (RS_middle_, *p* < 0.001; CS_middle_, *p* = 0.014; LS_apical_, *p* = 0.002).

**Fig. 2.**
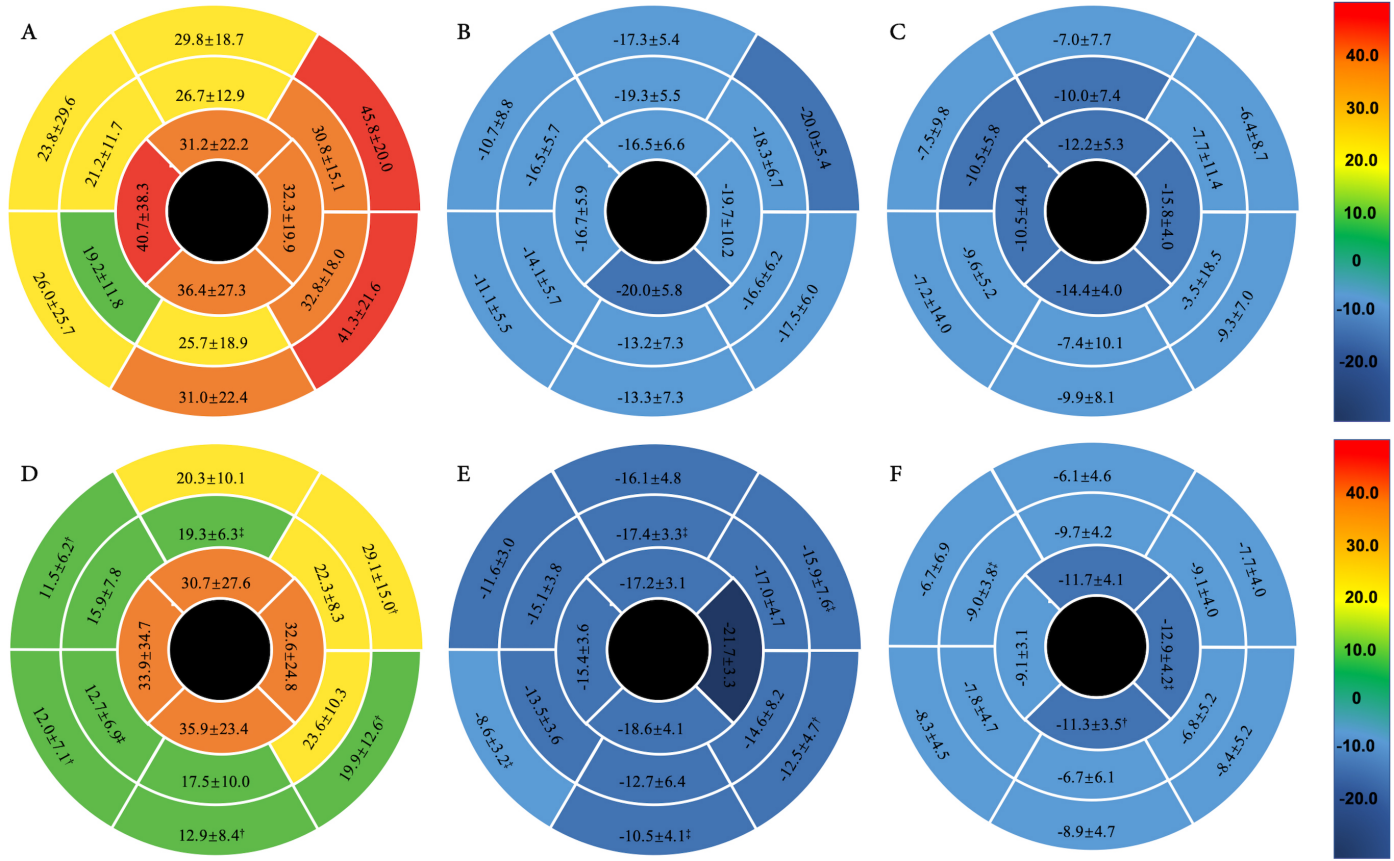
**The polar maps of myocardial strain values for the 16 segments were analyzed in subgroups of survivors (n = 59) and non-survivors (n = 16) with AL-CA**. (A–C) illustrate the standard deviations of the mean 16 segmental strain values, including radial strain, circumferential strain, and longitudinal strain for survivors, while (D–F) illustrate the standard deviations for non-survivors. Statistical comparisons reveal significant differences († *p* < 0.01 survivors versus non-survivors, ‡ *p* < 0.05 survivors versus non-survivors). AL-CA, light-chain cardiac amyloidosis.

**Fig. 3.**
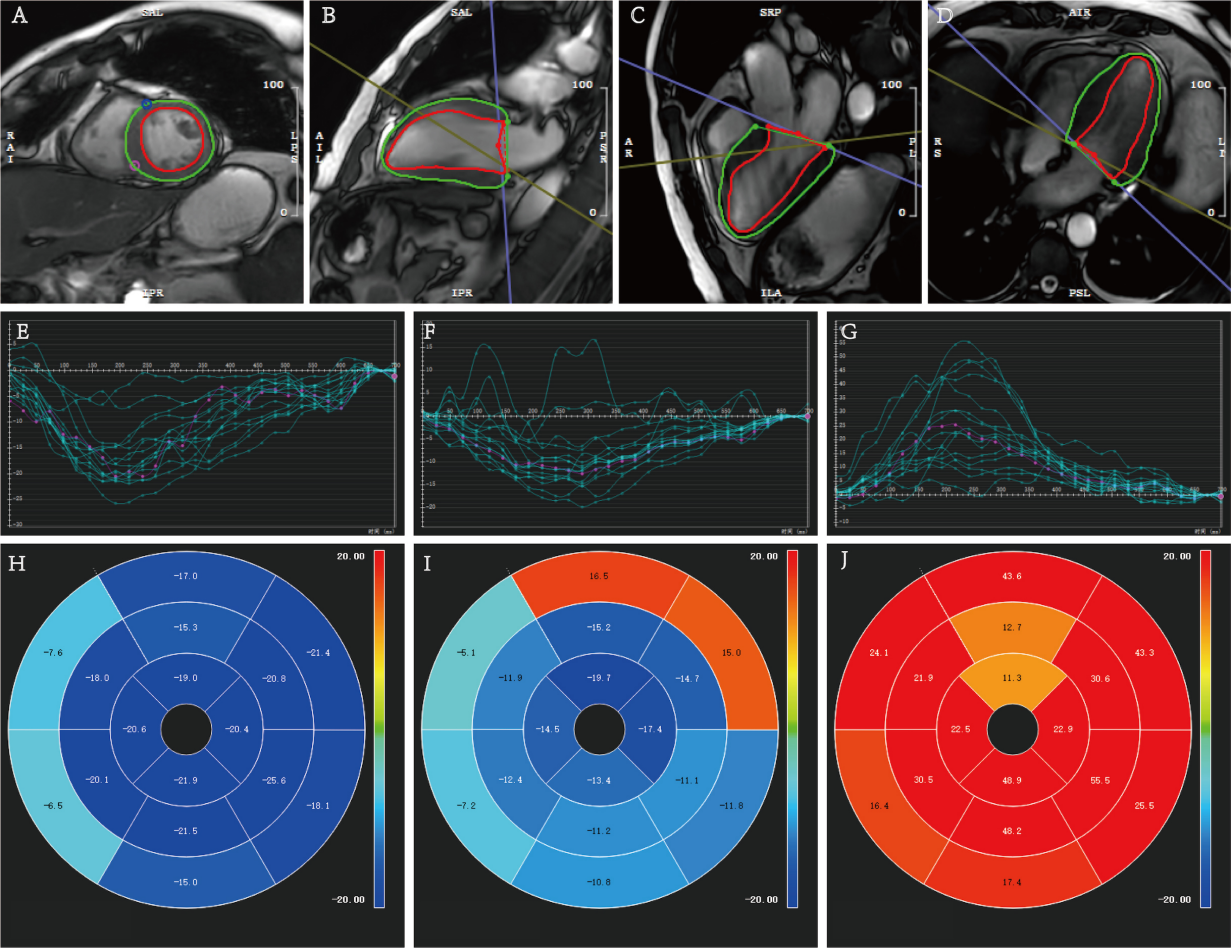
**Quantitative measurements of myocardial strain analysis in a 63-year-old male patient with confirmed AL-CA who died after a survival time of 14 months**. (A–D) Cardiac cine images illustrating the delineation of left ventricular endo- and epicardial contours (green represents epicardium, red represents endocardium) were obtained in short and long axes (two-, three-, and four-chamber views). (E–G) Myocardial strain curves of circumferential strain (CS), longitudinal strain (LS), and radial strain (RS). (H–J) The corresponding bull’s eye plots of the 16 myocardial segments. AL-CA, light-chain cardiac amyloidosis.

**Fig. 4.**
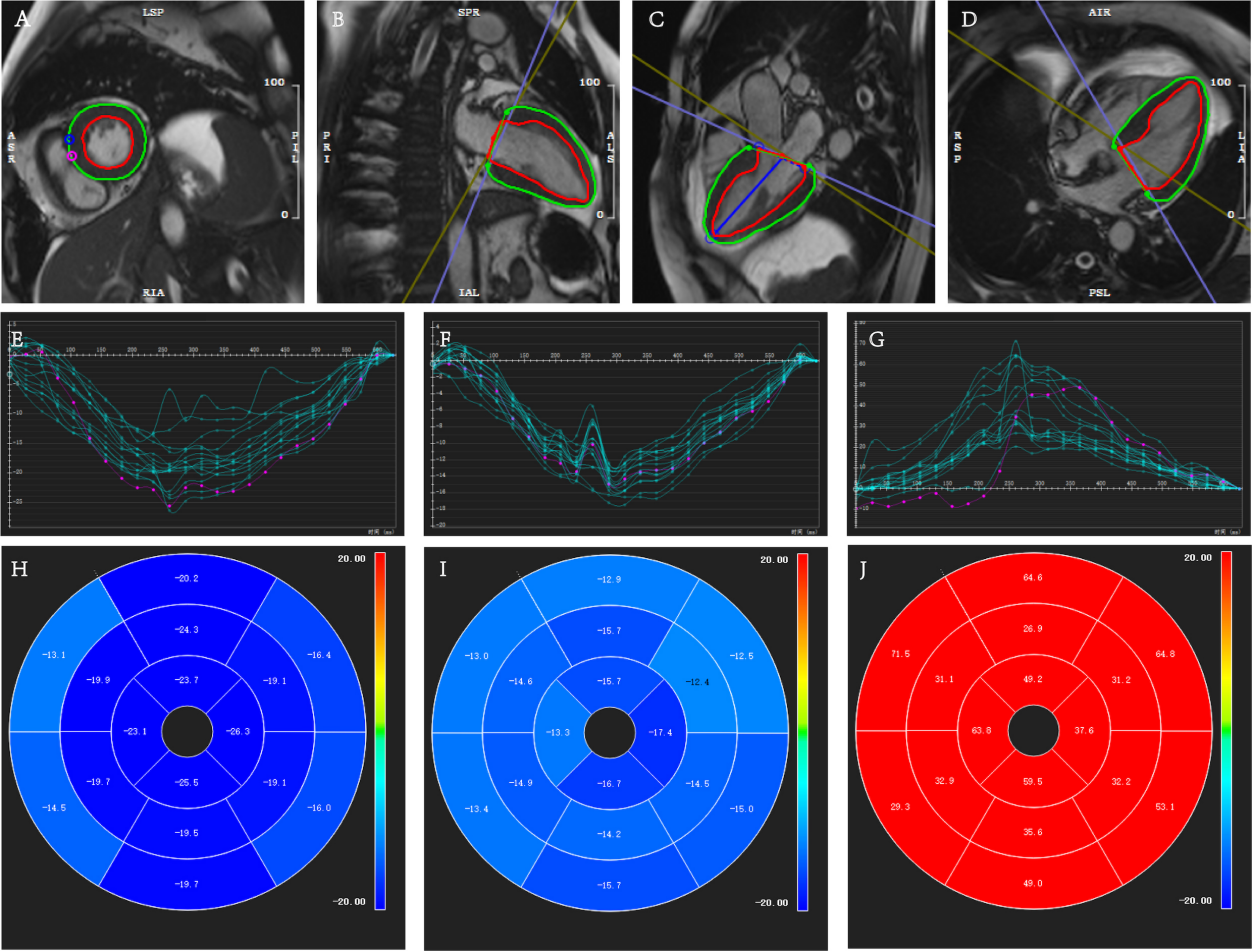
**Quantitative measurements of myocardial strain analysis in a 56-year-old female patient with confirmed AL-CA who was alive after a survival time of 28 months**. (A–D) Cardiac cine images illustrating the delineation of left ventricular endo- and epicardial contours (green represents epicardium, red represents endocardium) were obtained in short and long axes (two-, three-, and four-chamber views). (E–G) Myocardial strain curves of circumferential strain (CS), longitudinal strain (LS), and radial strain (RS). (H–J) The corresponding bull’s eye plots of the 16 myocardial segments. AL-CA, light-chain cardiac amyloidosis.

Fig. 2 illustrates the left ventricular strain parameters across the 16 myocardial segments defined by the American Heart Association (AHA). Noteworthy distinctions were identified in radial strains, specifically in the basal anteroseptal (*p* = 0.004), inferoseptal (*p* = 0.002), lateral (*p* < 0.001), inferolateral (*p* < 0.001), and anterolateral segments (*p* = 0.001). Furthermore, variations in radial strains were significant in the middle anterior (*p* = 0.011) and inferoseptal segments (*p* = 0.037). Examining circumferential strains revealed significant disparities in the basal inferoseptal (*p* = 0.020), lateral (*p* = 0.011), inferior (*p* = 0.003), anterolateral segments (*p* = 0.029), as well as in the middle anterior segments (*p* = 0.044). Additionally, longitudinal strains exhibited significant differences in the basal inferoseptal (*p* < 0.001), middle anteroseptal (*p* = 0.034), apical inferior (*p* = 0.006), and apical lateral segments (*p* = 0.014). Representative CMR acquisitions of myocardial strain from survivors and non-survivors groups in our cohort are illustrated in Figs. 3,4.

### 3.3 Survival Analysis

For survival analysis, two distinct approaches were employed (Table 2). Initially, clinical information and global strain parameters were considered, followed by a specific examination of the 16-segment strains.

**Table 2.** **Univariate and multivariate analyses of all patients (n = 75) between the survivors and non-survivors groups**.

	Global and segmental							16 myocardial segments					
	Univariate			Multivariate				Univariate			Multivariate		
Characteristics	Hazard ratio	95% CI	*p*-value	Hazard ratio	95% CI	*p*-value	Characteristics	Hazard ratio	95% CI	*p*-value	Hazard ratio	95% CI	*p*-value
BNP (pg/mL)	1.001	1.001–1.002	<0.001	1.001	1.001–1.002	<0.001	BNP (pg/mL)	1.001	1.001–1.002	<0.001	1.001	1.001–1.002	<0.001
Troponin I (µg/L)	0.618	0.160–2.387	0.485				Troponin I (µg/L)	0.618	0.160–2.387	0.485			
MDRD (mL/min/1.73 m^2^)	0.983	0.965–1.000	0.054				MDRD (mL/min/1.73 m^2^)	0.983	0.965–1.000	0.054			
LVEF (%)	0.945	0.899–0.994	0.028				LVEF (%)	0.945	0.899–0.994	0.028			
BU stage	3.522	1.480–8.382	0.004				BU stage	1.480–8.382	3.522	0.004			
RS_global_ (%)	3.391	1.087–10.571	0.035	0.225	0.057–0.887	0.033	RS_bas-anteroseptal_ (%)	35.442	0.360–3490.402	0.128			
CS_global_ (%)	0.115	0.015–0.872	0.036				RS_bas-inferoseptal_ (%)	3.927	1.257–12.266	0.019			
LS_global_ (%)	0.314	0.100–0.984	0.047				RS_bas-inferior_ (%)	10.087	2.242–45.374	0.003	0.168	0.035–0.803	0.025
							RS_bas-inferolateral_ (%)	6.700	1.515–29.632	0.012			
							RS_bas-anterolateral_ (%)	5.782	1.631–20.492	0.033			
							RS_mid-anterior_ (%)	5.125	1.639–16.030	0.005			
							RS_mid-inferoseptal_ (%)	8.962	1.175–68.363	0.034			

Note: BNP, B-type natriuretic peptides; MORD, ﻿Modified Diet in Renal Disease; BU, Boston University; LVEF, left ventricular ejection fraction; RS, radial strain; CS, circumferential strain; LS, longitudinal strain; CI, confidence interval.

The univariate Cox regression analysis identified several predictors of all-cause mortality, including BNP levels, BU stage, LVEF, and various global strain indices from the CMR assessments. In the subsequent multivariate Cox analysis, which included BNP, BU stage, LVEF, RS_global_, CS_global_, and LS_global_ (all *p* < 0.05), both BNP (hazard ratio: 1.001; 95% CI: 1.001, 1.002; *p* < 0.001) and RS_global_ (hazard ratio: 0.225; 95% CI: 0.057, 0.887; *p* = 0.033) remained significant independent predictors of all-cause mortality.

Expanding the analysis to include the 16-segment strain measurements of RS, such as RS_bas-inferoseptal_, RS_bas-inferor_, RS_bas-inferolateral_, RS_bas-anterolateral_, RS_mid-anterior_, and RS_mid-inferoseptal_, further underscored their individual importance as predictors of all-cause mortality in AL amyloidosis patients. In the multivariate Cox analysis incorporating predictors with *p* < 0.05 from the univariate Cox regression, both BNP (hazard ratio: 1.001; 95% CI: 1.001–1.002; *p* < 0.001) and RS_bas-inferior_ (hazard ratio: 0.168; 95% CI: 0.035–0.803; *p* = 0.025) emerged as significant independent predictors of all-cause mortality.

**Fig. 5.**
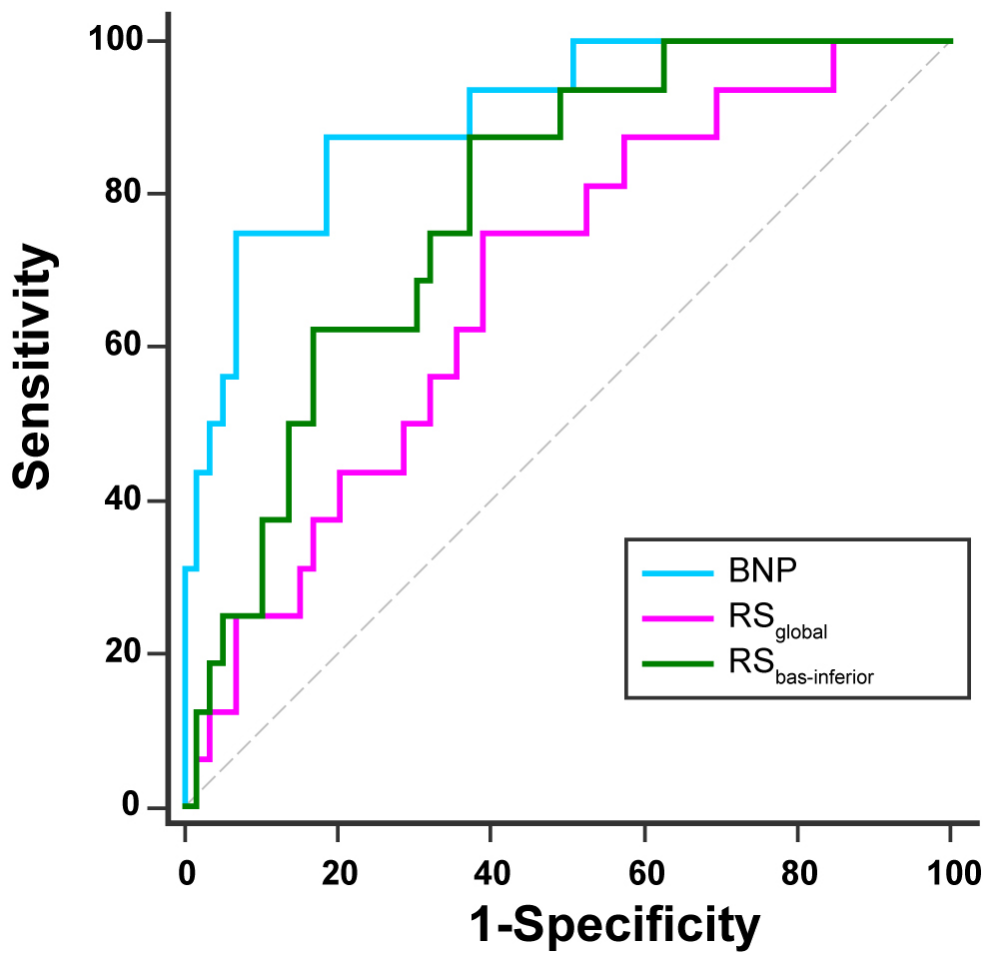
﻿**Receiver operating characteristic (ROC) curves for B-type natriuretic peptides (BNP), global radial strain (RS_𝐠𝐥𝐨𝐛𝐚𝐥_), and radial strain in the basal inferior segment (RS_bas-inferior_) for the endpoint**.

**Fig. 6.**
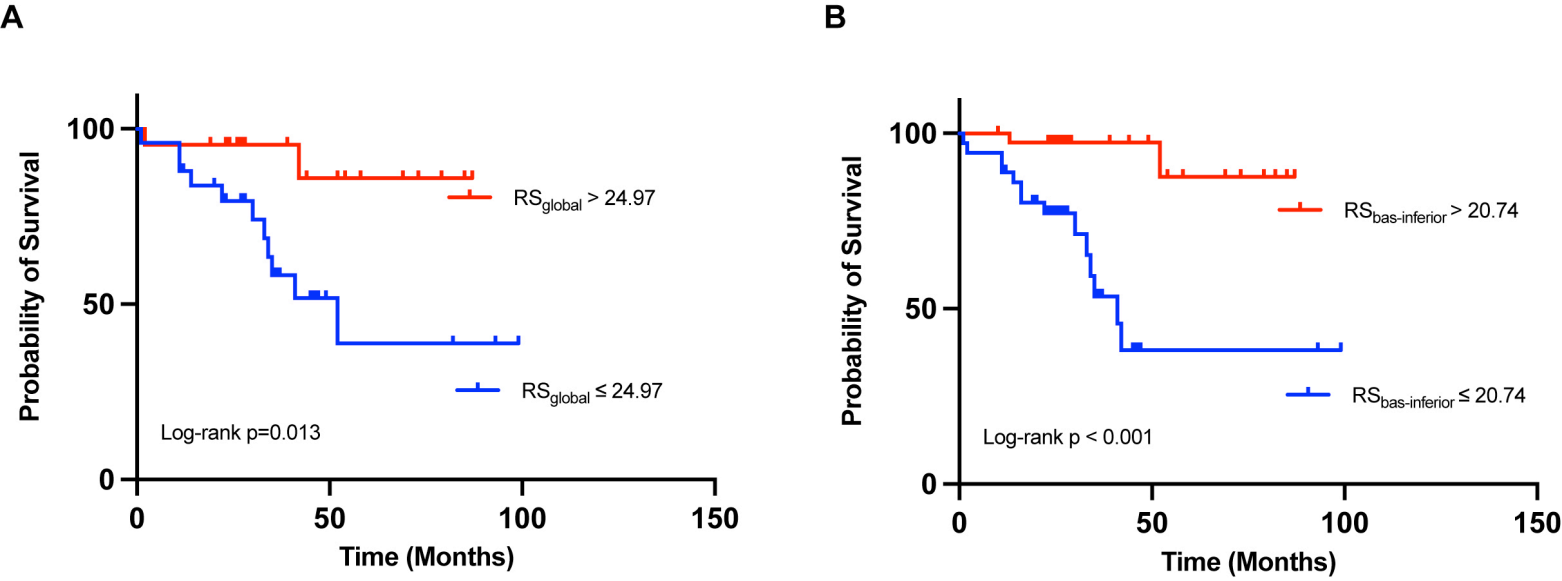
**Kaplan-Meier curves for event occurrence stratified by radial strain parameters**. Kaplan–Meier estimations of event occurrence over time, analyzed in relation to two specific parameters: global radial strain (RS_global_) with an optimized cut-off of 24.97% (A) and radial strain in the basal inferior segment (RS_bas-inferior_) with an optimized cut-off of 20.74% (B) for the endpoint.

In the ROC curve analysis (Fig. 5), BNP demonstrated the highest discriminatory ability (AUC = 0.901, ﻿95% confidence interval (CI): 0.811, 0.958) when comparing survivors and non-survivors. RS_global_ exhibited an AUC of 0.681 (95% CI: 0.563, 0.784), while RS_bas-inferior_ had an AUC of 0.786 (95% CI: 0.676, 0.872). Significant differences were found between the fields under the ROC curves for BNP and RS_global_ and BNP and RS_bas-inferior_ (*p* = 0.002, *p* = 0.029, respectively). Stratification was applied to RS_global_ and RS_bas-inferior_, with cut-off values of 24.97% and 20.74%, respectively. Kaplan–Meier survival curves were constructed for RS_basal_ and RS_bas-inferior_ subgroups, revealing notable differences in survival probability. A low RS_global_ level (below the cut-off value of 24.97%) and a low RS_bas-inferior_ level (below the cut-off value of 20.74%) were associated with an increased risk of death (Fig. 6, log-rank *p* = 0.013, log-rank *p* < 0.001, respectively).

### 3.4 Inter- and Intraobserver Variability

Inter- and intraobserver variabilities of strain values were analyzed (Table 3), and reproducibility of the LV strain was relatively desirable. The LV strain was highly reproducible, exhibiting a high intraclass correlation (inter- and intraobserver intraclass correlation coefficient >0.80).

**Table 3.** **Intraobserver and interobserver reproducibility for strain parameters (n = 20)**.

	Interobserver			Intraobserver		
	Mean difference ± SD	ICC	95% CI	Mean difference ± SD	ICC	95% CI
RS_global_	–1.22 ± 5.11	0.86	0.68–0.94	–0.62 ± 4.01	0.92	0.81–0.97
RS_basal_	–1.10 ± 4.55	0.88	0.73–0.95	0.56 ± 4.10	0.91	0.79–0.96
RS_middle_	–0.27 ± 3.36	0.93	0.82–0.97	–0.56 ± 1.84	0.98	0.94–0.99
RS_apical_	1.96 ± 7.32	0.83	0.62–0.93	–4.80 ± 8.24	0.82	0.54–0.93
CS_global_	–0.31 ± 1.56	0.92	0.82–0.97	0.14 ± 1.14	0.96	0.90–0.98
CS_basal_	–0.93 ± 1.72	0.89	0.70–0.96	0.30 ± 1.57	0.93	0.83–0.97
CS_middle_	0.05 ± 1.56	0.93	0.84–0.97	0.24 ± 0.62	0.99	0.97–1.00
CS_apical_	–0.35 ± 2.55	0.84	0.64–0.93	0.32 ± 1.62	0.93	0.84–0.97
LS_global_	–1.12 ± 1.46	0.82	0.42–0.94	0.39 ± 1.60	0.86	0.69–0.94
LS_basal_	–0.89 ± 2.04	0.80	0.56–0.92	–0.94 ± 1.88	0.82	0.58–0.93
LS_middle_	–1.41 ± 3.64	0.84	0.63–0.93	–0.94 ± 3.86	0.87	0.71–0.95
LS_apical_	–0.86 ± 1.10	0.88	0.54–0.96	0.85 ± 1.56	0.82	0.56–0.93

Note: RS, radial strain; CS, circumferential strain; LS, longitudinal strain; SD, standard deviation; ICC, intraclass correlation coefficient; CI, confidence interval.

## 4. Discussion

This study presents a comprehensive assessment of left ventricular remodeling and the prognostic value of left ventricular strains using the CMR-FT algorithm in an AL-CA patient cohort. Our investigation of myocardial strain in AL amyloidosis patients has yielded several significant findings. Firstly, patients with AL-CA showed significant reductions in radial strains and increases in circumferential and longitudinal strains observed in the basal myocardial segments when comparing survivors to non-survivors. Secondly, impairment in left ventricular myocardial mechanics was observed across various myocardial segments, with particular prominence in the septum and lateral wall. Thirdly, the radial strain in the global and the basal inferior segment exhibited potential as noninvasive markers for independently predicting all-cause mortality in AL amyloidosis patients.

The early detection and accurate classification of LV dysfunction in patients with AL-CA are crucial for predicting prognosis and determining appropriate therapeutic strategies to improve survival rates and quality of life [23]. Currently, cardiac serum biomarkers such as NT-proBNP, BNP, and cTnI are commonly used in clinical practice to assess the prognosis of patients with cardiac involvement [5]. CMR has emerged as the preferred diagnostic tool for identifying cardiac amyloidosis and establishing its association with mortality [24]. Previous studies have demonstrated the diagnostic and prognostic value of CMR techniques, such as late gadolinium enhancement (LGE), native T1 mapping, and extracellular volume (ECV) measurement in patients with amyloidosis [25, 26, 27, 28]. However, these techniques, such as LGE and ECV, may have limitations, particularly in patients with renal function impairment, which has frequently been observed in AL amyloidosis due to kidney involvement [29]. Additionally, contraindications for contrast agents further restrict their utility. CMR-FT is a user-friendly technique that can be applied by standard CMR cine SSFP sequences without requiring specialized acquisition or complex post-processing, demonstrating excellent reproducibility [30, 31]. Wan *et al*. [12] proposed that the LV strain may be used to monitor the extent of myocardial amyloid burden and may offer independent prognostic information for all-cause mortality in patients with AL amyloidosis. Previous studies have primarily focused on examining the prognostic implications of the global strain in myocardial amyloidosis. However, the current study represents a novel contribution by exploring the prognostic significance of both the global and segmental strains.

In patients who reached the study endpoint, notable alterations in myocardial strain patterns were observed, characterized by reduced radial strain coupled with increased circumferential and longitudinal strains in the basal myocardial segments. These findings corroborate existing research [12]. Moreover, distinctions in radial and circumferential strains at the apical segments between survivors and non-survivors were relatively minimal, except for in longitudinal strains. It is evident that basal segments are more susceptible to involvement, and the changes in strains in these segments may serve as an early indication of amyloid infiltration [32, 33]. These findings are consistent with a previous study by Li *et al*. [34], which demonstrated greater LV wall thickness in the basal and mid-cavity segments compared to the apex in patients with CA, indicating a higher degree of amyloid deposition in the basal segments and suggesting a possible underlying pathophysiology for early dysfunction in these regions. Intriguingly, significant disparities in segmental strains across the 16 myocardial segments were identified between the two groups, with a pronounced emphasis on the septal and lateral walls, necessitating further exploration of the underlying mechanisms.

Prior research has also shown that left ventricular LS_global_ and CS_global_, derived using CMR, have prognostic value for adverse events in patients with AL amyloidosis [12, 25]. In our study, which had a relatively long follow-up period (median, 34 months), BNP, RS_global_, and RS_bas-inferior_ exhibited independent associations with all-cause mortality. BNP is a recommended biomarker for risk assessment in AL amyloidosis, and our findings are consistent with previous studies [35]. In the Kaplan–Meier curve analysis used in our study, the curve for BNP did not exhibit statistical significance. RS_global_, reflecting myocardial fiber deformation toward the heart’s central cavity, correlated with changes in myocardial fiber thickness [36]. The novel contribution of this study lies in the potential utility of RS_bas-inferior_ for risk stratification in AL amyloidosis patients. This suggests that the lateral wall might be particularly susceptible to experiencing early or intensified pathological structural and functional changes. Nonetheless, the comprehensive mechanistic understanding of this phenomenon remains to be elucidated. Consequently, it is important to highlight that the measurement of RS_bas-inferior_ remains valuable for routine assessment of AL-CA prognosis.

Our study has several acknowledged limitations. Firstly, it was a single-center study, potentially limiting the generalizability of the findings. The limited number of patients enrolled and the relatively low incidence of all-cause mortality may impact our results. Secondly, the diagnosis of CA lacked confirmation through endomyocardial biopsy, potentially excluding patients in early disease stages without detectable left ventricular hypertrophy by echocardiography. However, our study’s inclusion criteria align with clinical diagnostic standards [37]. Thirdly, because some patients could not complete enhanced CMR examinations, we did not explore the correlation between myocardial deformation abnormalities and the specific location and quantity of amyloid protein accumulation. This correlation could be evaluated using advanced structural CMR techniques such as LGE and extracellular volume measurements [38]. Fourth, cardiac amyloidosis affects all four chambers; while previous studies have reported on this, our study did not investigate the prognostic value of strains in the other chambers; thus, this will be extensively analyzed in future studies [18, 39, 40]. Finally, the limited deformation in LV basal segments or global impairment of myocardial strain indices may not always directly reflect intrinsic myocardial dysfunction. It could be influenced, at least partially, by chest shape factors or artifacts, which should be considered, especially in individual cases [41].

## 5. Conclusions

Left ventricular radial strain in both the global and basal inferior segments demonstrates significant potential as noninvasive markers for independently predicting all-cause mortality in patients with AL amyloidosis when assessed using CMR-FT. Specifically, reduced global radial strain strongly correlates with adverse outcomes, reflecting overall myocardial function dysfunction. Additionally, reduced radial strain in the basal inferior segment is particularly noteworthy due to its unique association with myocardial involvement in AL amyloidosis. These findings underscore the value of incorporating detailed strain analyses into routine clinical evaluations, as they offer crucial prognostic information.

## Availability of Data and Materials

The data are obtainable at the request of the corresponding author in this study. They are not publicly available due to privacy issues.

## Supplementary Material

Supplementary material associated with this article can be found, in the online version, at https://doi.org/10.31083/j.rcm2511400.
