# Supplementary material for: Prognostic Value of Left Ventricular Myocardial Strain Parameters Derived from Cardiac Magnetic Resonance Feature Tracking Technique in Light-Chain Cardiac Amyloidosis Patients: A Pilot Study
Source: Rev Cardiovasc Med. 2024 Nov 14;25(11):400. doi: 10.31083/j.rcm2511400 (PMC11607509; doi:10.31083/j.rcm2511400)
Supplement: Supplementary file 1 [file 2153-8174-25-11-400-s1.docx]

**Supplementary Table 1**. MR imaging parameters of SSFP cine images.

|  | PHILIPS Ingenia (3.0 T) | | | | GE MR750 (3.0 T) | | | | SIEMENS MAGNETOM Prisma (3.0 T) | | | | SIEMENS MAGNETOM Aera (1.5T) | | | |
| --- | --- | --- | --- | --- | --- | --- | --- | --- | --- | --- | --- | --- | --- | --- | --- | --- |
|  | SA | 2-ch | 3-ch | 4-ch | SA | 2-ch | 3-ch | 4-ch | SA | 2-ch | 3-ch | 4-ch | SA | 2-ch | 3-ch | 4-ch |
| TR (ms) | 2.9 | 2.8 | 2.8 | 2.8 | 3.4 | 3.6 | 3.7 | 3.4 | 3.3 | 3.3 | 3.3 | 3.3 | 2.6 | 2.7 | 2.7 | 2.7 |
| TE (ms) | 1.4 | 1.4 | 1.4 | 1.4 | 1.5 | 1.6 | 1.7 | 1.5 | 1.43 | 1.43 | 1.43 | 1.43 | 1.11 | 1.16 | 1.16 | 1.16 |
| FA (°) | 45 | 45 | 45 | 45 | 45 | 45 | 45 | 45 | 80 | 80 | 80 | 80 | 80 | 80 | 80 | 80 |
| FOV (mm^2^) | 350×350 | 350×350 | 350×350 | 350×350 | 380×380 | 320×320 | 320×320 | 380×380 | 340×265 | 340×284 | 340×284 | 340×284 | 340×340 | 340×276 | 340×276 | 340×276 |
| Thickness (mm) | 10 | 8 | 8 | 8 | 8 | 8 | 8 | 8 | 8 | 6 | 6 | 6 | 8 | 6 | 6 | 6 |
| Spacing (ms) | 0 | 0 | 0 | 0 | 0 | 0 | 0 | 0 | 1.6 | 1.2 | 1.2 | 1.2 | 2 | 1.2 | 1.2 | 1.2 |
| Voxel (mm^3^) | 1.8×1.8×10 | 2×2×8 | 2×1.8×8 | 2×1.8×8 | 1.7×1.7×8 | 1.4×1.4×8 | 1.4×1.4×8 | 1.7×1.7×8 | 1.6×1.6×8 | 1.6×1.6×6 | 1.6×1.6×6 | 1.6×1.6×6 | 1.8×1.8×8 | 1.8×1.8×6 | 1.8×1.8×6 | 1.8×1.8×6 |

Note – SSFP, steady-state free precession, SA, short axes, ch, chamber, TR, repetition time, TE, echo time, FA, flip angle, FOV, field of view.
